# Bias in the Machine? A Solution to the Isolationist Problem Through a Sociotechnical Understanding of Bias in AI Ethics

**DOI:** 10.1007/s11948-026-00612-0

**Published:** 2026-07-25

**Authors:** Gabriela Arriagada-Bruneau

**Affiliations:** Instituto de Éticas Aplicadas, Instituto de Ingeniería Matemática y Computacional, Pontificia Universidad Católica de Chile, Santiago, 4860 Chile

**Keywords:** AI ethics, Technosolutionism, AI bias, Sociotechnical systems, Bias management

## Abstract

Dominant approaches to bias in artificial intelligence (AI) are structured by what I identify as the isolationist problem: the tendency to treat bias as a discrete, technically addressable flaw within the AI development pipeline, rather than as a relational phenomenon embedded in social, institutional, and political arrangements. This problem is sustained by two mutually reinforcing orientations: technocentrism, which reframes ethical challenges as engineering problems amenable to computational resolution, and the bias-centric conception of fairness, which reduces fairness to statistical mitigation and obscures its contested, context-dependent character. Together, these orientations produce ontological, epistemic, and practical forms of narrowing ethical imagination and channel intervention into technically tractable but socially limited responses. Against this, I propose sociotechnical sensitivity as both an analytical orientation and a normative commitment: a sustained attentiveness to the ways in which AI systems are constitutively embedded in social relations, institutional arrangements, cultural norms, and power structures. The paper’s central argumentative shift is to change the narrative from bias mitigation to bias management—treating bias not as a defect to be corrected but as an ongoing condition to be governed. These arguments are developed through an extended analysis of the well-known case of the COMPAS algorithm, a recidivism risk-assessment prediction tool, illustrating three practical axes of bias management: contextualisation, institutionalisation, and iteration. The gap between sociotechnically sensitive AI ethics and its realisation is ultimately a matter of governance design and political will, not merely a problem of missing methods or tools.

## What Do We Call Bias in AI?

Bias in AI is commonly described as systematic discrepancies in system behaviour that lead to disadvantageous outcomes for particular individuals or groups (Mehrabi et al., 2021; Ntoutsi et al., 2020). These discrepancies may originate in skewed training data, modelling choices, or human decisions embedded across the development pipeline. Crucially, such sources rarely operate independently; they interact in ways that reproduce and intensify one another.

A frequent typology distinguishes between societal, technical, and cognitive biases. Societal biases reflect entrenched inequalities—such as those related to race, gender, or class—embedded in historical data and institutional norms (Ntoutsi et al., 2020; Roselli et al., 2019). Technical bias arises from system design, including data preprocessing, model architecture, and evaluation procedures. Even apparently neutral design choices, such as training on demographically narrow datasets, can produce models that fail to generalise (Rajpurkar et al., 2022). Cognitive biases stem from the reasoning patterns of developers and users, including confirmation bias or anchoring, which may shape how data are selected, labelled, and interpreted (Soleimani et al., 2021, 2022).

These categories often overlap. Schwartz et al. (2022) highlight how human, social, and computational biases, are distributed across the AI lifecycle. Arriagada-Bruneau (2024) even recognises these categories as cognitive, technical, and social biases underscoring a central point: bias is not merely a technical glitch but a sociotechnical phenomenon (Arriagada-Bruneau, 2024). The persistence of bias becomes especially visible in mechanisms such as proxy variables and feedback loops. Removing protected attributes from a dataset does not prevent discrimination if correlated features remain; postcode, for instance, may function as a proxy for race due to patterns of residential segregation. Likewise, system outputs can reshape user behaviour, reinforcing disparities over time. What appears as neutral optimisation can thus contribute to cumulative exclusion.

Bias may also arise from the internal dynamics of machine learning itself. Deep learning systems frequently converge on different local minima during training, producing models that generalise unevenly—often disadvantaging underrepresented groups (Schwartz et al., 2022). Even with balanced data, the structure of optimisation can yield persistent performance gaps. While bias—aware techniques may mitigate such effects, the complexity of these systems makes complete elimination improbable.

Therefore, here we will understand bias in AI not as a discrete defect that can be removed once and for all, but as a layered and evolving phenomenon that must be understood relationally and contextually.

### Technocentrism in AI

To introduce the isolationist problem of bias in AI, it is first necessary to examine the technocentric assumptions that have shaped its emergence. Technocentrism refers broadly to the belief that technological solutions are inherently effective—and often superior—in addressing complex social, political, and ethical challenges. In the context of AI, this belief is not typically articulated as a formal theoretical position, but manifests implicitly in the practices of technology corporations and developers, particularly those prioritising scalability and optimisation. These practices tend to foreground data-centric efficiency while systematically sidelining normative, social, and contextual dimensions. As Peeters et al. (2021, p. 219) note:

Although followers of techno-centrism admit that new technologies can introduce additional problems, they are also eager to point out that these problems can again be solved by applying additional technology.

This circular logic reinforces the notion that AI’s ethical and social challenges require not cultural or political interventions, but increasingly in datasets (Lloyd, 2018), label bias in annotation (Jiang & Nachum, 2019), or inductive bias in algorithm design (Wilson & Frank, 2023). While these diagnoses are valid, the technocentric solution (unsurprisingly) is more engineering—better algorithms, cleaner data—rather than questioning the structural conditions that generate inequality associated to those biases in the first place.

As a result, ethical problems are reframed as technical puzzles. This outlook marginalises areas of study such as sociology, philosophy, science and technology studies, and political theory that offer richer accounts of AI’s entanglement with power. It also excludes affected communities from deliberation, particularly those most impacted by algorithmic harms. Ultimately, technocentrism promotes a depoliticised model of ethics, one that privileges metrics over meaning and treats bias as a quantifiable error rather than a symptom of systemic injustice.

A growing body of scholarship has drawn attention to the limits of this perspective. In the healthcare domain, Gichoya et al. (2022, 2023) critique the reliance on statistical measures—such as true positive and false negative rates—as proxies for fairness in medical AI. While technically informative, such metrics often fail to capture the complex socio-clinical contexts in which these tools operate. Moreover, they highlight how financial incentives, rather than clinical relevance, frequently drive AI adoption. Bui and Noble (2020) examine public backlash, the so-called “techlash”, against Silicon Valley’s dominance, driven by concerns over surveillance, discrimination, and democratic erosion. They argue that many corporate efforts to develop “ethical AI” remain trapped within a technocentric frame, seeking to *perfect* rather than interrogate technological development:

The goal is to perfect or ‘unbias’ the technology, rather than account for the asymmetrical power relationships and gravity of history that renders the development and deployment of such projects deeply uneven, unethical, and even immoral. (Bui & Noble, 2020, p. 166)

Similar critiques have emerged in the regulatory sphere. Regine Paul (2022), building on Coglianese and Lai (2022) and Krafft et al. (2022), shows how the drive to “remove human bias” through AI often masks deeper debates about regulatory priorities and value pluralism. She warns that the “bias obsession” in AI policy sidelines questions about whose interpretations are rendered policy-relevant and whose are excluded. This, she argues, privileges corporate risk management over broader concerns like redistribution and justice (cf. Niklas & Dencik, 2021, 2024; Padden & Öjehag-Pettersson, 2021).

Fjeld et al. (2020), in a comprehensive analysis of 36 AI ethics guidelines, further identify a “potential technochauvinism:” the assumption that technical fixes, e.g., improved datasets and algorithmic tuning, can sufficiently address systemic discrimination. While such recommendations are often well-intentioned, they rarely question the institutional and societal conditions from which bias emerges:

[…] many documents point to biased data […] as the source of discrimination and unfairness in AI, but few recognise the role of human systems and institutions. […] This reflects a potentially technochauvinistic orientation—the idea that technological solutions are appropriate and adequate fixes to the deeply human problem of bias and discrimination. (Fjeld et al., 2020, pp. 47–48)

The term *technochauvinism*, popularised by Broussard (2018), complements the critique of technocentrism. Where the latter reflects an overarching belief in technological progress, the former denotes an overconfidence in the superiority of computational solutions over human judgement. Broussard argues that what is needed is not the wholesale rejection of AI, but a careful, context-sensitive evaluation of *when* technology is and is not the right tool for the task. This demands hybrid epistemologies that respect human expertise and local knowledge alongside algorithmic modelling. Yet, as Eubanks (2018) and Green (Green, 2021, 2022) argue, technical “fixes” often obscure the very structural conditions such as poverty, racism, or exclusion, that generate the harms AI seeks to solve.

In sum, technocentrism and its associated logic of technosolutionism isolate ethical concerns from their political and historical contexts, reducing complex social problems to questions of optimisation. This has profound implications for how bias is conceptualised and addressed in AI systems. By treating bias simply as a technical error, developers often remain within a closed loop of computational refinement. Ethical questions are reframed as algorithmic challenges; power asymmetries are translated into metrics; and justice is rendered legible only in terms of statistical parity^1^.

To break out of this loop, I argue that a shift is needed in how ethics is embedded within AI ecosystems. This requires a *sociotechnical sensitivity*, one that recognises AI as embedded within social, cultural, and economic systems and resists the instinct to treat every ethical concern as a problem to be debugged. But to make this shift, first, we need to clarify the conceptual ambiguity surrounding AI bias.

## Conceptual Ambiguity and the “Bias-Centric” View of Fairness in AI

In addition to the influence of technocentric solutionism on how AI developers perceive and address bias, a further complication arises from the conceptual ambiguity that surrounds key ethical terms in the field. It is worth specifying that this ambiguity is present in how academic literature in the AI ethics debate define certain concepts, and it is not an ambiguity in a technical sense, e.g., AI training data.

Whittlestone et al. (2019), in their influential roadmap for research on the ethical and societal implications of AI, data, and algorithms, identify this ambiguity as a major obstacle to effective interdisciplinary engagement. They argue that:

[…] the ambiguity of many central concepts currently used to identify salient issues […] like ‘fairness’ […]. While they have served to highlight common themes emerging from case studies, many of these terms are overlapping and ambiguous. This stems partly from the fact that different fields, disciplines, sectors, and cultures can use these concepts in substantially different ways, and partly from inherent complexities in the concepts themselves. As a result, discussions of the ethical and societal impacts of ADA risk being hampered by different people talking past each other. (Whittlestone et al., 2019, p.14)

This ambiguity not only undermines interdisciplinary communication but, as I argue, contributes to a reductive orientation that privileges technical definitions and solutions over context-sensitive understandings.

### A Conflation of “Bias” and “(Un)Fairness”

A salient example of this ambiguity lies in the conflation of terms such as bias and fairness. As Whittlestone et al. (2019) explain, the term bias is used in divergent ways across academic and professional domains. In statistics, for instance, a “biased sample” refers to one that does not accurately represent the target population. This is a descriptive, non-normative use. In contrast, in psychology, law, and the social sciences, bias often implies unjust or prejudicial treatment, carrying a strong normative charge.

This difference is not merely semantic; it can lead to profound misunderstandings in practice. A dataset may be deemed “unbiased” in statistical terms, yet still reflect deeply embedded social prejudices:

a dataset which is ‘unbiased’ (in the statistical sense) may nonetheless encode common biases (in the social sense) towards certain individuals or social groups. [Therefore], distinguishing these different uses of the same term is important to avoid cross-talk. (Whittlestone et al., 2019, p. 15)

This cross-talk can easily impede efforts to address ethical concerns in AI, since stakeholders may assume they are in agreement when, in fact, they are working with fundamentally different understandings of core concepts. As the authors further elaborate, various disciplines tend to approach complex terms like fairness in distinct ways:

[Q]ualitative social scientists would often seek to highlight the rich differences in how different stakeholders understand the concept. Similarly, philosophical ethicists often seek to highlight inherent dilemmas and in-principle problems for different definitions of a concept, whereas many lawyers and researchers from other policy oriented disciplines would look for operational definitions that are good enough to resolve in-practice problems. (Whittlestone et al., 2019, p. 15)

Such disciplinary variation also contributes to conceptual instability. This is especially problematic when terms with distinct meanings—such as bias, fairness, and discrimination—are used interchangeably in both academic literature and policy documents, often to refer to overlapping yet non-identical phenomena. Whittlestone et al. identify this as a key dimension of conceptual ambiguity, noting that “the terms ‘bias’ and ‘fairness’ are often conflated, with some discussions of such cases simply defining bias as unfair discrimination” (2019, p. 16).

While I concur with their broader argument, I argue for further refinement in their critical standpoint of this conceptual ambiguity. It is the conflation of bias *and unfairness*, rather than bias and fairness per se, that warrants scrutiny. Typically, bias is conceptualised as a deviation from accuracy or neutrality, either as a human cognitive flaw or a technical error within a system. Unfairness, however, is a normative judgement: it refers to the presence of unjust or illegitimate disadvantage, often arising from structural or institutional arrangements.

The failure to maintain a conceptual distinction between these terms gives rise to what I refer to as the bias- centric view of fairness.

### A Bias-Centric View of Fairness

As I mentioned, a prevalent issue in contemporary AI development is the bias-centric view of fairness—the tendency to treat *bias* as the primary, and often sole, indicator of fairness or unfairness in AI systems. Under this perspective, fairness is effectively reduced to the technical task of bias mitigation: if algorithmic bias is identified and minimised, typically through statistical or computational methods, then fairness is presumed to have been achieved (to some extent).

This reductionist framing is particularly evident in public and professional discourses surrounding AI. In academic conferences, social media, and policy forums, one frequently encounters arguments that position bias or fairness as a strictly mathematical problem, disconnected from political or ethical concerns. Within this view, fairness is often defined in terms of statistical parity, such as the divergence between conditional probability distributions (e.g., comparing height distributions of men and women using maximum likelihood estimation or kernel density estimation). If the divergence equals zero, the system is considered unbiased and thus, fair; if it is non-zero, it is labelled unfair.

Such formulations exemplify the technocentric reductionism embedded in the bias-centric approach. Fairness is rendered synonymous with statistical equivalence, while deeper questions about representation, power, and structural injustice are sidelined (Aler Tubella et al., 2022; Bartneck et al., 2021; Bui & Noble, 2020). Even when statistical parity is defended in the name of diversity, these arguments frequently lack engagement with normative accounts of fairness or the perspectives of those directly affected by algorithmic decisions.

This perspective is problematic for several reasons. First, it ignores the fact that fairness is a contested and context-dependent ethical concept that cannot be exhaustively defined or resolved through mathematical formalism. Second, it neglects dimensions of justice—such as recognitional, procedural, and distributive fairness—which are not readily captured by statistical metrics. Third, it marginalises alternative epistemologies and modes of engagement, particularly those rooted in lived experience, community participation, or sociopolitical critique. Ultimately, the bias-centric view facilitates a conceptual conflation: bias becomes equated with unfairness, and fairness becomes limited to what can be computationally optimised. This creates a closed loop in which technical definitions of fairness dictate what counts as bias, rather than allowing ethical theory or affected communities to shape those definitions. As a result, fairness is reframed as a technically “fixable” property of systems, rather than as a multidimensional goal requiring institutional, social, and political consideration.

In short, while bias mitigation remains an important aspect of AI ethics, the bias-centric paradigm, I argue, is both inadequate and potentially harmful. It narrows the scope of fairness to what is technically tractable, obscuring the broader pursuit of justice, equity, and inclusivity. A more robust engagement with fairness must move beyond bias reduction to interrogate the structural and normative frameworks within which AI systems are designed, deployed, and evaluated.

Jamie Brandon (2021) warns of the ethical risks associated with such a narrow focus on bias mitigation. In particular, he critiques the tendency to prioritise model “de-biasing” over the reflective evaluation of systemic patterns and harms. Brandon argues that efforts to eliminate bias within a model may suppress valuable insights into underlying social injustices:

Debiasing a model censors out insights around unethical bias. Analysts need to be able to find these insights and share them with management, peers, and their society. Evidence shows transparency with biases instigates change. By choosing not to debias the model, the analyst will be more aware of problems that exist. […] Before finding a solution, awareness and a proper understanding of the problem are key. (Brandon, 2021, p. 107)

Brandon’s point is that models reflecting biased outcomes can, paradoxically, serve as diagnostic tools. Rather than concealing flaws through premature technical fixes, it may be more productive to surface and examine these patterns to promote social awareness and institutional change. In this sense, debiasing efforts that lack contextual reflection risk dulling the critical edge of ethical inquiry.

The persistence of a bias-centric view of fairness is frequently rooted in, or reinforced by, a technocentric mindset—even when not explicitly acknowledged. This framing fosters the illusion that mitigating bias is equivalent to achieving ethical AI. In this regard, the work of Johnson and Verdicchio (Johnson & Verdicchio, 2024, 2023) provides a compelling critique through their concept of the “addition fallacy.” This fallacy denotes the mistaken belief that ethical considerations—such as post hoc fairness metrics or bias mitigation algorithms—can be added to a technological system in a way that renders it ethical or socially responsible. The fallacy stems from a failure to recognise the ontological disjunction between computational systems and normative ethical reasoning. It reflects a form of *moral add-on logic*, in which the augmentation of technology with ethical components is mistaken for substantive ethical transformation. This is a misconception we must resist.

Rejecting the bias-centric paradigm is thus essential for a meaningful integration of ethics into AI development. As Hooker (2021) argues, bias mitigation alone does not constitute ethical engagement. Effective mitigation requires a deep understanding of the origins of bias and an awareness of the limits of technical intervention. She highlights two demanding preconditions for so-called “data fixes” (1): advance knowledge of which sensitive attributes are responsible for harm, and (2) complete and reliable labelling of those attributes across datasets—conditions that are rarely met in practice. And, even when these conditions are met, AI systems may still infer and act upon proxy variables, inadvertently reinforcing disparities through indirect mechanisms. Hooker argues that addressing harm requires more than data adjustments; it demands scrutiny of model architectures, design decisions, and the broader socio-technical systems in which AI is embedded.

In sum, the bias-centric approach to fairness misconstrues fairness as a computational objective and sustains the technocentric illusion that ethical adequacy can be engineered. Yet, a robust notion of fairness cannot be achieved through statistical parity or mitigation techniques *alone*.

As Degelin et al. (2024) demonstrate, bias in AI systems is not simply the result of imbalanced training data. Rather, it is reproduced and intensified through fine-tuning processes and model-layer interactions with social identity cues such as race and gender. Their study of BERT-based grant recommendation systems revealed persistent normative bias against Black principal investigators (PIs), even when datasets were balanced and stripped of explicit identity markers. Intriguingly, the inclusion of PI names during fine-tuning amplified bias, despite apparent demographic parity. Moreover, efforts to correct underrepresentation often yielded inconsistent or counterproductive outcomes, for example, Asian and Hispanic PIs received disproportionately lower-valued recommendations following dataset balancing. These findings reinforce a key point: bias in large language models is not confined to discrete features or data points but is distributed across model architectures and learning dynamics, making it resistant to modular or localised interventions.

Bias is embedded not only in what models learn, but in *how* they generalise.

## The Isolationist Approach to AI Bias, and Why We Should Call this a *Problem*

Building on the critiques against technocentrism, the bias-centric view of fairness, and the challenge of conceptual ambiguity surrounding bias and fairness, I now turn to the “isolationist approach” to bias in AI. This critique emerged from two literature reviews conducted as part of the development of the Bias Network Approach (BNA) by Arriagada-Bruneau et al. (2025). As presented by the authors, this approach is presented as a pervasive tendency to treat bias as a discrete, localisable, and technically remediable defect within the AI development process. Under this approach, bias emerges *inside* datasets, models or optimisation routines and can therefore be corrected through improved engineering: better-curated data, fairness constraints, algorithmic adjustments, or hyperparameter tuning. What is “isolated” in this conceptualisation is both the bias itself, and more importantly the complex web of social, institutional and political relations through which bias is both generated and reinforced. Hence, narratives and discourses prevailing in AI contexts (both academic and in the industry) operate with the fundamental idea that bias can be “eliminated” and that bias mitigation takes care of fairness issues in AI. Although this is, arguably true, this does not tell the whole story.

**Fig. 1:**
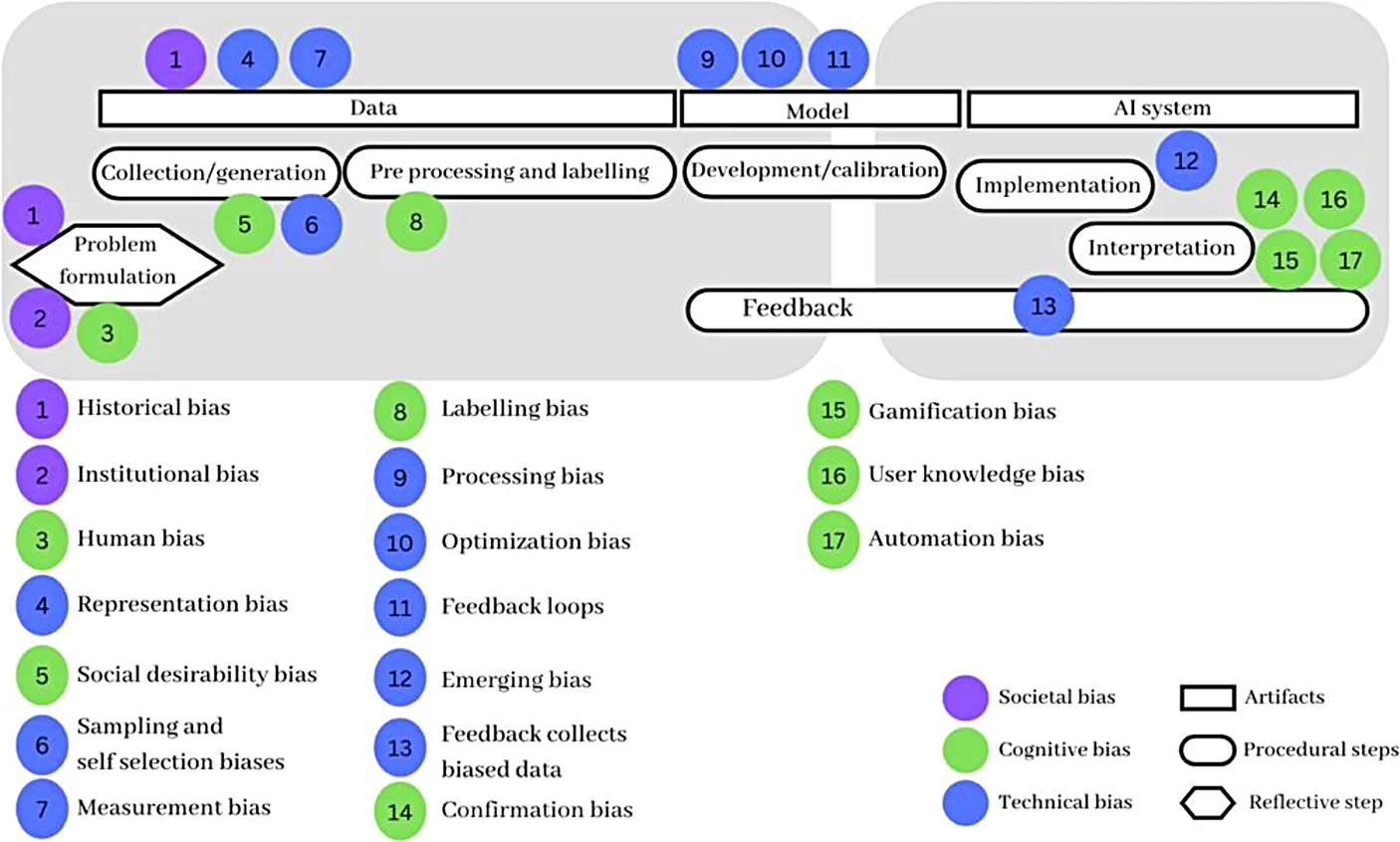
Biases as often mentioned in the literature, placed in different stages of the AI pipeline (Arriagada-Bruneau et al., 2025)

The authors map the most commonly cited types of bias onto the AI development pipeline. Societal and historical biases tend to cluster in early-stage processes like data collection and problem scoping, while cognitive and design-related biases appear during model development. Technical biases, such as measurement error or selection bias, are associated with data pre-processing and algorithmic tuning. This mapping evidences a compartmentalised treatment of bias, see Fig. 1:

Furthermore, their analysis showed that associated mitigation strategies techniques to address these biases such as dataset balancing, label reweighting (e.g., SMOTE), and adversarial debiasing, while often methodologically robust, they rarely interact with the underlying institutional or cultural structures from which bias originates. For instance, oversampling underrepresented groups in facial recognition datasets may correct statistical imbalance, but it does not confront the systemic exclusion that shaped the dataset to begin with.

Crucially, as the authors notice, the literature remains heavily skewed towards pre-deployment stages, with limited attention paid to post-deployment dynamics, user interaction, or feedback mechanisms (Feuerriegel et al., 2020; Kizilcec & Lee, 2022). This reflects an assumption that bias is a flaw to be engineered out, rather than an evolving property of sociotechnical systems in use. The isolationist framing thus contributes to “ethical myopia”, with this I refer to an overreliance on technical fixes that overlook how bias is co- produced across layers of design, implementation, and interpretation.

Whilst agreeing with the analysis of Arriagada-Bruneau et al. (2025), I will call this phenomenon the isolationist *problem* of AI bias instead of an “approach”, because it not only implies adopting a heavily technocentric and a bias-centric fairness approach, but it also implies that bias is often abstracted from the institutional histories, organisational routines, socioeconomic hierarchies and policy decisions that shape both the *construction* of training data and the *contexts of deployment* in which AI systems operate. As a result, bias is detached from the very conditions that give it ethical salience, even in cases where the link seems evident, such as historical bias. Historical bias, for example, is usually defined as “a result of pre-existing individual and societal or group biases transferred into training datasets” (Foka et al., 2025, p.5839), however solutions that mitigate this bias often imply that this can be understood as a technical anomaly—a glitch to be debugged—rather than as a manifestation of deeper structural inequalities and sociopolitical arrangements that can either crawl back into the AI ecosystem (not just its training phase, but also its deployment and feedback stages), or can be kept alive or reintroduced by humans in the loop.

A helpful reviewer questioned whether the phenomenon described here genuinely warrants the designation of an isolationist problem, suggesting it might more parsimoniously be understood as a constituent element of a broader technocentric orientation. This observation warrants a clarification of why the term is not merely stylistic but carries distinct conceptual weight.

The label *isolationist problem* is employed because it is intended to capture a specific analytical move that both precedes and underpins technocentric solutionism: the treatment of bias as an isolated property of technical artefacts, rather than as a relational phenomenon embedded within sociotechnical systems. Technocentrism, by contrast, describes a disposition towards technological forms of intervention; the isolationist problem, on the other hand, names a prior conceptual reduction in how the problem itself is constituted - one that may, of course, facilitate and reinforce that technological disposition. Calling it a problem signals that bias is detached from the institutional histories, social structures, and political dynamics through which it is produced and sustained and is instead reconstituted as a bounded technical defect, an instance amenable to isolation, correction, and resolution, thereby rendering the system “fair” in the sense implied by bias-centric conceptions of fairness. The label therefore draws attention to an ontological and epistemic narrowing that renders technocentric responses appear both natural and sufficient. In this sense, the *isolationist problem of AI bias* names not simply one approach among others, but a structural limitation inscribed within prevailing framings of AI bias.

This isolationist problem can be further characterised, and I propose it has three interconnected dimensions worth considering:

- **Ontological isolation:** bias is treated as a property of technical artefacts (datasets, models, parameters), rather than as a relational phenomenon that surfaces through the dynamic interplay of human actors, institutional practices and material constraints across the AI lifecycle.
- **Epistemic isolation:** the knowledge deemed necessary to understand and address bias is assumed to be primarily technical. This marginalises insights from social sciences, humanities, law and the lived experiences of communities affected by algorithmic systems, privileging algorithmic metrics over contextual interpretation and critical enquiry. This epistemic narrowing mirrors broader critiques in the literature that computational fairness metrics obscure the social dynamics and power asymmetries that produce inequality in the first place.
- **Practical isolation:** interventions emphasise discrete technical corrections—data augmentation, fairness-aware learning, or threshold adjustments—while leaving institutional practices and power structures that generate and perpetuate inequitable outcomes largely intact. The implicit assumption is that once the technical biases are mitigated, the system will function fairly in the world; what is overlooked are the socio-institutional conditions that shaped both the data and the deployment environment.

The consequence of the isolationist problem of AI bias, I argue, is that ethical imagination becomes constrained by what is technically feasible rather than what is socially just. When bias is framed exclusively as an internal glitch, the scope of acceptable solutions contracts to algorithmic adjustment, dataset curation, and optimisation of fairness metrics. In this sense, isolationism does more than misdescribe the nature of bias; it channels ethical effort into technically tractable but socially limited forms of response. Ethical concerns become synonymous with technical challenges, power asymmetries are translated into numbers on a confusion matrix, and justice is made legible only through statistical parity or unweighted loss functions.

The isolationist problem, because it is closely tied to a broader technocentric orientation within AI development and governance, it provokes ethical issues to be routinely framed as problems *within* the technology, rather than *about* how technology interacts with social reality. Hence the tendency of adopting an isolationist approach to bias in AI is symptomatic of a deeper tendency: bias is compartmentalised as a pipeline issue and responded to with narrowly tailored engineering responses, sidelining the very contexts that make bias ethically urgent.

Finally, why is the isolationist approach, in fact a problem and not just an approach as initially called by Arriagada-Bruneau et al. (2025)? Because its limitations are not simply methodological preferences but normatively and epistemically distorting. An approach becomes a problem when it systematically constrains understanding in ways that undermine the very goals it purports to advance. In this case, isolating bias within the technical components of AI systems misrepresents its ontological status as a relational and socially embedded phenomenon, narrows the epistemic resources deemed relevant for its interpretation, and restricts practical interventions to those that are technically tractable rather than socially transformative. The result is not a neutral framing among alternatives, but a structurally biased orientation that obscures causal responsibility, deflects attention from institutional and political drivers of inequality, and legitimises partial solutions as if they were comprehensive. In this sense, isolationism is not just incomplete; it is ethically consequential, because it shapes which harms are rendered visible, which actors are held accountable, and which forms of change are considered possible.

Thus, to overcome the isolationist problem of AI bias, a shift is needed in how ethics is embedded within AI ecosystems. We need an approach that recognises AI as deeply embedded within social, cultural and economic systems and resists the instinct to treat every ethical concern as a problem to be debugged. The following sections examine how developing a *sociotechnical sensitivity* that goes beyond a sociotechnical understanding of AI systems, can help reframe and respond to bias in ways that are both ethically robust and practically meaningful.

## The Importance of Adopting and Developing a Sociotechnical Sensitivity

Before proceeding, a terminological clarification is in order. Throughout this paper, the term *sociotechnical sensitivity* is used consistently to denote both a foundational analytical disposition and the operationalised normative practice it entails. As an analytical orientation, sociotechnical sensitivity refers to the capacity to perceive and attend to the embeddedness of technical systems within social, institutional, and political arrangements—recognising that AI systems are never merely technical objects but always components of broader sociotechnical assemblages. As a normative practice, it denotes a sustained, reflexive attunement to how those arrangements shape and are shaped by AI systems, and a commitment to treating that entanglement as analytically and ethically central rather than as contextual background noise. The two dimensions are inseparable: the disposition to perceive sociotechnical entanglement is what motivates the commitment to responding to it. This integrated conception is what distinguishes sociotechnical sensitivity from mere sociotechnical awareness and from narrowly technical approaches to AI ethics.

Accordingly, a clear consequence of failing to recognise the isolationist problem and reflecting a lack of sociotechnical sensitivity of AI bias is what Morozov (2014) has termed *solutionism*, which Nachtwey and Seidl (2024) have more recently extended to *solutionist ethics*. Under this paradigm, complex social and political problems are reframed as technical glitches amenable to algorithmic resolution. Ethical deliberation is reduced to procedural compliance, and political concerns such as justice, power, or redistribution, which are bracketed in favour of performance metrics and statistical parity. The intention here is not to dismiss technical mitigation altogether; such tools remain essential for addressing concrete harms. However, when they become the primary or exclusive mode of ethical engagement, they risk obscuring the deeper structural inequalities that shape AI systems. In such cases, ethics becomes an exercise in technical refinement rather than a critical reflection on the societal implications of computational decision-making.

The isolationist problem also promotes a false sense of completeness. Once a particular form of bias has been mitigated, say, through debiasing word embeddings or equalising error rates, there is a tendency to consider the fairness problem resolved. Yet fairness is not a terminal state but an ongoing condition. It concerns not only what can be measured or statistically corrected, but what is valued, who decides, and whose interests are served, dimensions that shift across different stages of development and deployment. By treating fairness as a fixed outcome achievable through discrete interventions, an isolationist approach forecloses more ambitious conversations about justice, redistribution, and systemic change. The appeal of such methods lies in their specificity and apparent objectivity, e.g., attributes that align well with the epistemic preferences of technical disciplines. But when they are not integrated into a broader ethical and contextual analysis, they risk producing systems that are procedurally fair yet substantively unjust.

To overcome these limitations, I propose a sociotechnical reframing of how we approach AI bias, one embedded in what I have called *sociotechnical sensitivity*, that is, an orientation that locates bias not as an anomaly within a discrete system, but as a reflection of the broader environments in which that system is conceived, developed, and deployed. This distinction is consequential: it is what makes it meaningful to speak of *managing* bias rather than merely *mitigating* it. Mitigation addresses symptoms: management requires attending to the conditions that generate and sustain them.

Sociotechnical sensitivity, hence, denotes a sustained attentiveness to the ways in which technical systems are constitutively entangled with social relations, institutional arrangements, cultural norms, and power structures. It is not simply an acknowledgement that context matters—a point that has become something of a commonplace in AI ethics discourse—but a methodological and normative commitment to treating that entanglement as analytically central. To be sociotechnically sensitive is to resist the conceptual move that separates the technical from the social, the artefact from its conditions of production, or the algorithm from the organisational and political contexts in which it operates. It requires asking not only what a system does, but who shaped it, under what conditions, in whose interests, and with what consequences for different communities and across different temporal horizons.

This sensitivity has both a descriptive and a normative dimension. Descriptively, it orients analysis towards the sociotechnical assemblages within which AI systems are embedded, attending to the institutional histories, labour relations, epistemic hierarchies, and governance arrangements that condition how systems are built and how they perform. Normatively, it insists that ethical evaluation cannot be confined to the technical layer of a system but must extend to these broader conditions. As West et al. (2019) have rightly observed:

As the focus on AI bias and ethics grows, the scope of inquiry should expand to consider not only how AI tools can be biased technically, but how they are shaped by the environments in which they are built and the people that build them. (p. 6)

Sociotechnical sensitivity also has an important temporal dimension. A sociotechnically sensitive approach resists the tendency to evaluate AI systems at a single point in time, typically at deployment, and instead emphasises an understanding of bias and fairness as dynamic properties that evolve across the lifecycle of a system and in relation to shifting social contexts. The normative consequences of AI systems are rarely foreseeable at the design stage; ethical engagement must therefore be ongoing, anticipatory, and reflexive rather than confined to a post hoc audit or a one-time fairness assessment.

This stands in productive dialogue with recent work on value dynamics in AI. Kudina and van de Poel (2024) argue that AI systems should be understood not as autonomous artefacts but as elements of sociotechnical systems in which technological development co-evolves with human values and institutional practices. On their account, values are not fixed norms awaiting translation into code but are emergent and situated within socio-material contexts:

[…] values have a dual nature: on the one hand, they serve as moral orienteers in problematic situations, giving guidance on how to resolve them, and on the other, they are themselves a product of these human-technological practices, which give rise to them, can challenge their fitness or propose new values. As such, values, and by extension the moral problems and moral opportunities, are a dynamic property that materialises within a complex system of human- technology-world dimensions and across these dimensions. (Kudina & van de Poel, 2024, p. 5)

This account of value dynamics reinforces the argument for sociotechnical sensitivity: if the values at stake in AI systems are themselves emergent and contested, then no static technical specification of fairness can be adequate. Ethical design must remain open-ended, iterative, and responsive to the shifting normative terrain in which AI systems operate.

Developing a sociotechnical sensitivity therefore challenges the assumption that ethics can be adequately addressed through post hoc technical fixes or normative frameworks. It instead requires participatory and iterative design processes that engage with context and affected communities from the outset. Related work on embedded ethics and contextual fairness similarly emphasises that AI evaluation must attend to institutional, political, and cultural conditions (Green, 2021).

A sociotechnically sensitive approach calls, in particular, for the inclusion of those most likely to bear the costs of biased or unjust systems, communities that are frequently marginalised from the design process and from the epistemic frameworks that govern what counts as a problem in the first place.

This last point connects sociotechnical sensitivity to questions of power and political economy. Addressing AI bias is not only a technical task but a political one, involving questions of historical marginalisation, epistemic authority, and structural inequality. Drawing on feminist epistemology, Draude et al. (2019) frame bias as relational and embedded in structures of power, emphasising positionality and epistemic plurality as conditions of responsible design. In this light, fairness is not a universal metric but a contested, context- dependent value (D’Ignazio & Klein, 2020) -one whose meaning cannot be settled by technical consensus alone but requires ongoing democratic negotiation.

Ultimately, sociotechnical sensitivity foregrounds the recognition that technologies do not merely implement pre-existing values but actively reshape moral landscapes, creating new possibilities, foreclosing others, and altering the very terms in which ethical questions are posed and, in this case, the contexts in which we evaluate AI biases. Addressing AI bias therefore requires sustained moral engagement within evolving sociotechnical systems, and not the mere application of sociotechnical definitions or frameworks, but a continuous, reflexive practice attuned to the ways in which the technical and the social are always mutually constitutive.

## From Bias Mitigation to Bias Management: Sociotechnical Sensitivity in Practice

### The Persistence of Bias and the Limits of Mitigation

Before turning to the question of how AI developers might operationalise a sociotechnical understanding of bias, it is worth confronting a foundational point that the mitigation paradigm consistently obscures: bias, in the broad sociotechnical sense developed throughout this paper, is not a problem that can be solved. It can be tracked, interpreted, redistributed, and governed but not eliminated. This is not a mere sign of defeat or resignation; it is epistemological and ethical realism. AI systems are trained on data generated by historically unequal social processes, deployed into institutions shaped by structural asymmetries, and evaluated according to criteria that themselves carry normative assumptions. No technical procedure can fully decouple a system from its conditions of production. The question is therefore not whether bias will persist, but how developers, institutions, and affected communities can engage with its manifestations responsibly and continuously.

This reframing is consequential for practice. A mitigation mindset structures development around thresholds, points at which bias has been sufficiently reduced to warrant deployment. A management mindset structures development around ongoing responsibility, the recognition that deployment is not a terminal point but an entry into a dynamic sociotechnical environment whose moral stakes evolve over time. The shift from mitigation to management is thus not a call for vaguer ethics, but for a different mode of intervention: one that treats bias as a dynamic property of sociotechnical systems rather than a static defect in models, a lens of sociotechnical sensitivity.

This distinction becomes concrete when examined through a paradigmatic case. The remainder of this section therefore centres its analysis on COMPAS (Correctional Offender Management Profiling for Alternative Sanctions), a risk-assessment tool widely discussed in academic literature. This case is chosen not because it is exceptional, but because it is well-documented, much-contested, and precisely the kind of high-stakes domain in which the inadequacy of purely technical responses to bias is most visible. The analysis that follows uses COMPAS to illustrate how a sociotechnical sensitivity to bias management operates along three practical axes: contextualisation, institutionalisation, and iteration.

### COMPAS and the Inadequacy of Technical Mitigation

COMPAS assigns recidivism risk scores used by judges in bail, sentencing, and parole decisions across multiple US jurisdictions. In 2016, ProPublica’s investigation (Angwin et al., 2016) reported that the tool produced substantially higher false positive rates for Black defendants, that is, it more frequently classified individuals as high risk who did not subsequently reoffend. While at the same time it generated higher false negative rates for white defendants. This asymmetry prompted extensive debate about what fairness in algorithmic decision-making requires, and on what grounds.

The subsequent dispute between ProPublica and the tool’s developers, Northpointe, is instructive. Both sides used technically defensible fairness criteria: ProPublica emphasised unequal error rates across racial groups; Northpointe maintained that the model was *calibrated*, meaning that a given score predicted roughly the same probability of rearrest regardless of race. As Chouldechova (2017) demonstrated, these two desiderata are mathematically incompatible when base rates differ across groups (as they do in any society characterised by structural racial inequality in policing). No technical adjustment can simultaneously satisfy both conditions. The disagreement is not, at its core, a technical dispute. It is a dispute about which conception of fairness ought to govern decisions about human liberty, and that dispute cannot be resolved by optimising a loss function.

From a mitigation perspective, this is a frustrating impasse. From a management perspective, it is precisely the expected outcome: fairness is a contested, context-dependent value and its definition in any given deployment context must be settled through processes of normative deliberation, not statistical selection.

### Contextualisation: Treating Data as Institutional Artefacts

The first axis of bias management is contextualisation: the practice of situating data, model design, and optimisation targets within the institutional and historical processes that produced them.

COMPAS does not predict criminal behaviour directly; it predicts rearrest. Rearrest is a policing outcome shaped by patrol density, surveillance priorities, and reporting practices that fall disproportionately on marginalised communities. What the model presents as a forecast of individual risk is, at least in part, a forecast of future institutional attention. The training data therefore encode prior enforcement priorities, not a neutral ground truth. Improving predictive accuracy under these conditions may deepen systemic inequity rather than reduce it: a more accurate predictor of rearrest is, under conditions of racially unequal policing, a more accurate predictor of racialised surveillance.

In practical terms, contextualisation requires that developers treat their datasets as historical and institutional artefacts before treating them as statistical objects. This involves forms of inquiry that are largely absent from standard machine learning pipelines: analysis of how data were generated, which populations are systematically under- or misrepresented, what institutional practices shaped what was recorded, and what categories carry embedded normative weight. In the COMPAS case, variables such as employment stability, residential history, and peer associations function as proxies for structural disadvantage. Removing explicit racial indicators from the model does not prevent it from reconstructing race through these correlated features, a phenomenon well-documented in the fairness literature (Barocas & Selbst, 2016).

Contextualisation therefore demands collaboration beyond the engineering team. Criminologists and sociologists can interpret how risk-relevant features are socially produced; legal scholars can clarify how score outputs interact with judicial discretion, plea bargaining, and sentencing norms; community organisations can surface how risk labels function in practice and whose interests they serve. This collaboration must begin at the problem formulation stage—not at the audit stage, where contextual concerns tend to be raised too late to reshape fundamental design decisions. Documentation practices must expand accordingly: beyond recording dataset provenance, development teams should document the social provenance of their data: the institutional practices, historical conditions, and structural inequalities that shaped what was measured and what was omitted.

### Institutionalisation: Embedding Bias Management in Organisational Structures

The second axis is institutionalisation: the embedding of bias management in organisational routines and governance structures rather than leaving it to the discretion of individual developers.

The COMPAS controversy illustrates that the most consequential decisions about a predictive system are not purely technical: they include which fairness criterion governs the model, how scores are communicated to judges, what caveats accompany outputs, and what recourse is available to those adversely affected. In the absence of institutional structures, these decisions are made implicitly and inconsistently, often by default. Bias management requires making them explicit and accountable.

Concretely, this involves several overlapping mechanisms. Cross-functional review bodies, with decision- making authority rather than merely advisory status, can ensure that normative and contextual concerns are institutionally heard before deployment. Structured documentation practices, covering not only technical specifications but the normative assumptions embedded in optimisation targets, the trade-offs accepted, and the dissenting views raised, create an auditable record of how decisions were made. Escalation procedures allow frontline users or affected parties to raise concerns when system outputs generate patterned disadvantage and ensure that such concerns reach decision-makers rather than being absorbed by technical teams as isolated anomalies.

Critically, COMPAS also demonstrates that institutional accountability must extend beyond pre- deployment validation. Risk scores influence judicial behaviour; judges may detain more individuals classified as high-risk, which disrupts employment and housing, elevating future risk indicators and altering the data distribution on which subsequent models are trained. The system thus participates in reshaping the very conditions it purports to predict. Bias management must therefore treat deployment as the beginning of governance, not its end. Organisations must monitor how risk scores affect sentencing patterns, detention rates, and downstream social outcomes, tracking not only model accuracy but second-order institutional effects: which individuals are deterred from applying for diversion programmes, which groups are most frequently subject to judicial override, and how frontline practitioners interpret and selectively discount model scores.

These requirements raise a crucial institutional question. In the case of COMPAS, the tool was produced by a private vendor. The scoring algorithm was proprietary, and access to its inner workings was restricted, a condition that made independent audit effectively impossible (Angwin et al., 2016). Bias management, in this context, is not merely a design challenge but a regulatory and political one. It requires governance frameworks that compel transparency, mandate independent review, and establish rights of contestation for those whose life outcomes are materially shaped by algorithmic scores.

### Iteration: Managing Bias as a Dynamic and Ongoing Achievement

The third axis is iteration: the recognition that bias is not a stable property of a fixed system but a dynamic outcome of an evolving sociotechnical assemblage.

A mitigation mindset assumes a relatively stable world in which bias can be reduced to acceptable levels and the system certified as fair. A management mindset assumes that both the system and its environment are continuously changing. In the COMPAS context, the influence of risk scores on judicial practice alters the caseload composition entering the system, modifying the base rates against which the model was calibrated. Shifts in policing priorities, legal reforms, or demographic change alter the social conditions the model encodes. Fairness, on this view, is not a property achieved at a point in time but an ongoing achievement that must be continually reasserted through monitoring, review, and revision.

Iteration therefore involves longitudinal sociotechnical impact assessment. This means an evaluative practice that extends well beyond pre-deployment testing and periodic retraining. It requires tracking how deployment redistributes opportunities, burdens, and institutional attention across social groups over time; examining how frontline practitioners adapt their behaviour in response to scores; and assessing whether appeal or contestation mechanisms are accessible in practice to those most affected. In domains such as criminal justice, where algorithmic outputs interact directly with state power over liberty, such mechanisms of accountability are not optional supplements to technical governance but essential conditions of legitimate deployment.

The iterative character of bias management also implies an important corollary: the possibility of withdrawal. If ongoing monitoring reveals that a system consistently deepens structural inequity despite iterative adjustment, as critics have argued is the case with predictive policing tools more broadly (Brayne, 2020; Richardson et al., 2019), then bias management must include the institutional capacity to suspend or discontinue use. A management framework that cannot recommend non-deployment is not genuinely accountable; it has merely institutionalised the appearance of governance.

### Redistributed Expertise and the Translational Role of the AI Developer

The three axes described above, contextualisation, institutionalisation, and iteration, share a common implication: they require forms of expertise and institutional practice that extend well beyond the remit of technical AI development as conventionally understood. Technical experts remain central, but they are no longer the sole arbiters of what bias is or how it should be addressed. Domain experts contribute knowledge of institutional goals and constraints; legal scholars clarify regulatory and rights-based implications; social scientists analyse structural inequalities and category formation; affected communities provide situated knowledge of lived harms and unanticipated consequences.

The role of the AI developer within this expanded framework becomes partly translational: rendering model behaviour legible to non-technical stakeholders while, in turn, translating normative and contextual concerns into design requirements, monitoring criteria, and governance procedures. This does not eliminate technical expertise; it repositions it within a broader accountability structure. Techniques such as reweighting, fairness constraints, or adversarial debiasing remain valuable tools for mitigating certain effects, but they become instruments within a governance process rather than endpoints. Their adequacy is judged not only by statistical parity but by how they interact with institutional practices and whether they reduce or merely displace harm.

Interdisciplinary collaboration of this kind is often messy and uncomfortable. Different disciplines operate with different standards of evidence, different timescales, and different normative commitments. Disagreement is not a failure of the process but an intrinsic feature of sociotechnical deliberation about contested values. Bias management therefore requires organisational tolerance for ambiguity and contestation, qualities that sit uneasily with the culture of technical optimisation and definitive solutions that characterises many AI development environments, but that are embraced through the lens of sociotechnical sensitivity practice.

The practices described above are demanding. They require sustained resources, institutional support, and, at times, the willingness to question whether a given system should be built or deployed at all. In commercial and governmental settings driven by efficiency, scale, and rapid deployment, these conditions are frequently absent. Bias management therefore confronts not only epistemic complexity but structural political and economic constraints.

Recognising these constraints is itself part of what bias management requires. It means acknowledging that developers operate within institutional structures that shape what is feasible and that meaningful engagement with bias may require organisational reform, regulatory intervention, or public scrutiny that extends beyond the technical team and, indeed, beyond the organisation. In this respect, the gap between the conceptual aspiration of a sociotechnical approach to bias and its practical implementation is not merely a matter of missing tools or insufficient methodology. It is a matter of political will and governance design.

Bias management is thus not a technical add-on to existing development pipelines, but a reorientation of AI development towards shared and institutionalised responsibility for social consequences. This does not ask developers to solve structural injustice. It asks that AI development be organised such that the social and institutional realities into which systems are introduced are treated as constitutive of system behaviour, rather than as externalities to be corrected, if noticed at all, after the fact.

## Conclusion

In this paper I have argued that dominant approaches to bias in AI are structured by a fundamental conceptual limitation: the isolationist problem. By treating bias as a discrete, technically remediable defect within the AI pipeline, prevailing frameworks systematically abstract it from the social, institutional, and political conditions that produce and sustain it. This is not simply a methodological preference among alternatives; it is, as I have stressed, an epistemically and normatively distorting orientation, one that constrains ethical imagination, narrows the scope of legitimate intervention, and renders partial solutions as if they were comprehensive ones.

The paper traced the architecture of this problem across three interconnected dimensions. First, technocentrism reframes ethical concerns as engineering challenges, marginalising the disciplinary and community-based forms of knowledge necessary for meaningful engagement with AI’s societal consequences. Second, the bias-centric conception of fairness reduces a contested, multi-dimensional ethical concept to a matter of statistical optimisation, producing the illusion that fairness can be engineered rather than negotiated. Third, the isolationist problem itself names the ontological and epistemic narrowing that underlies both tendencies: bias is reconstituted as a bounded defect, detached from the structural inequalities and institutional histories that give it ethical salience.

Against this, I have proposed sociotechnical sensitivity as both an analytical orientation and a practical commitment. This is not merely an acknowledgement that context matters, a point that has become increasingly common in AI mainstream narratives in the ethics discourse, but a sustained methodological and normative disposition that resists the separation of the technical from the social, the artefact from its conditions of production, and the algorithm from the institutional and political environments in which it operates. Sociotechnical sensitivity is what makes meaningful the shift from bias mitigation to bias management: from a developmental mindset oriented towards thresholds and terminal corrections, to one structured around ongoing responsibility, institutional accountability, and reflexive engagement with the evolving sociotechnical environments in which AI systems are embedded.

The analysis of COMPAS illustrated what this shift demands in practice. Contextualisation requires treating data as institutional and historical artefacts rather than statistical objects and beginning normative deliberation at the problem formulation stage rather than after deployment. Institutionalisation requires embedding bias management in organisational governance structures, with accountability mechanisms that extend beyond the technical team and survive beyond the moment of release. Iteration requires monitoring bias as a dynamic property of systems-in-use, attending to second-order institutional effects, and preserving the capacity for withdrawal when ongoing assessment reveals that a system deepens rather than reduces structural inequity.

These demands are neither modest nor easily satisfied. They require sustained resources, interdisciplinary collaboration, tolerance for ambiguity, and a willingness, often structurally disincentivised, to slow or halt development when social stakes are high. They also require recognition that developers operate within political and economic structures that shape what is feasible, and that meaningful bias management may therefore entail organisational reform, regulatory intervention, and public scrutiny extending well beyond any single development team.

This is, ultimately, the deeper implication of taking the isolationist problem seriously. The gap between the aspiration of a genuinely sociotechnical approach to AI bias and its practical realisation is not primarily a gap in methods or tools. It is a gap in governance design and political will that demands the enactment of the sociotechnical sensitivity. Bias management cannot be operationalised in environments that treat fairness as a compliance obligation to be discharged and forgotten, or in regulatory frameworks that accept technical audits as substitutes for structural accountability. Realising the potential of a sociotechnically sensitive approach to AI ethics requires institutional conditions that do not yet exist at scale, and advocating for those conditions is itself part of what such an approach demands.

The argument advanced here does not ask AI developers to resolve structural injustice. What it asks is more specific and, I would argue, more tractable: that the social and institutional realities into which AI systems are deployed be treated as constitutive of system behaviour rather than as externalities, and that the responsibility for managing bias be distributed across the interdisciplinary, organisational, and regulatory structures commensurate with its systemic character. Bias, understood in this way, is not a problem to be solved. It is a condition to be governed, and governing it well is a moral and political achievement that no algorithm, however carefully tuned, can accomplish alone.

## Data Availability

Not applicable.

## Footnotes

1 A well-known and documented case of this phenomenon is the case of the COMPAS algorithm used for predicting recidivism risk in the United States. See (Angwin et al., 2016; Dieterich et al., 2016; Lagioia et al., 2022; Washington, 2019).
